# In Vivo Two-Photon Imaging of Astrocytes in GFAP-GFP Transgenic Mice

**DOI:** 10.1371/journal.pone.0170005

**Published:** 2017-01-20

**Authors:** Dongjun Guo, Jia Zou, Nicholas Rensing, Michael Wong

**Affiliations:** Department of Neurology and the Hope Center for Neurological Disorders, Washington University School of Medicine, St. Louis, MO, United States of America; University of Nebraska Medical Center, UNITED STATES

## Abstract

Astrocytes play important roles in normal brain function and neurological diseases. In vivo two-photon excitation laser scanning microscopy has the potential to reveal rapid, dynamic structural changes in cells in a variety of physiological and pathological conditions. The type of in vivo imaging method has been shown to affect the plasticity of dendritic spines of neurons, but the optimal in vivo imaging methods of astrocytes have not been established. We compared open-skull and thinned-skull imaging methods for two-photon laser microscopy of live astrocytes in neocortex of GFAP-GFP transgenic mice. The thinned-skull method provided stable image intensity and morphological features of astrocytes in vivo over at least one week, with no evidence of astrogliosis. In contrast, the open-skull method resulted in significant changes in image intensity and induced astrogliosis. The thinned-skull method is the preferred approach for in vivo imaging of astrocytes under most conditions involving gross astrocyte modulation or causing astrogliosis.

## Introduction

Astrocytes, a subtype of glial cell in the central nervous system, are responsible for maintenance of homeostasis in the brain by regulating local ion concentrations, pH, energetic state, and metabolism [1]. In addition to housekeeping functions, astrocytes play more active physiological roles in brain signaling and synaptic plasticity [2]. Under pathological conditions, astrocyte dysfunction may also contribute to neurological disorders, such as stroke, traumatic brain injury, and epilepsy [3]. Astrodegeneration, gliosis, and other structural changes in astrocytes are often identified in pathological specimens from animal models and patients with epilepsy and other neurological disorders.

Novel imaging methods for studying astrocytes provide important insights into both the physiological and pathological roles of astrocytes in normal brain function and neurological diseases. In contrast to the fixed, static view provided by conventional pathological studies, in vivo two-photon excitation laser scanning microscopy (2PLSM) is an indispensable method to investigate dynamic changes in cellular and subcellular structure and function in live tissue [4,5]. Several recent studies utilizing in vivo time-lapse 2PLSM in the mouse brain demonstrate that astrocytes may undergo rapid, dynamic structural changes under a variety of physiological and pathological conditions [6,7,8,9]. However, the optimal imaging methods for in vivo imaging of astrocytes are not well-established.

Two primary methods with different merits and drawbacks have been widely used for in vivo cellular imaging of the brain, the open-skull and thinned-skull techniques [10,11,12,13]. The open-skull technique is achieved by performing a complete craniotomy to provide direct, unimpeded optical access to the brain. However, the open-skull method may cause direct irritation of the brain, leading to inflammation, astrogliosis, and higher dendritic spine turnover. Alternatively, the thinned-skull technique is achieved by thinning of the skull to the inner cortical bone without completely penetrating the skull or epidural space, potentially causing less perturbation or inflammation of the underlying brain. Comparisons of these two methods have been performed in analyzing in vivo imaging of neurons, particularly dendritic spines, in mouse cortex and have detected dramatic differences in spine motility between the two techniques [10]. However, no systematic comparison has been done between the two techniques in the observation of live astrocytes in vivo. In the present study, we refined and compared the open-skull and thinned-skull techniques with repeated 2PLSM for in vivo imaging of astrocytes in neocortex of GFAP-GFP transgenic mice.

## Materials and Methods

### Ethics statement

Care and use of animals were conducted according to an animal protocol approved by the Washington University School of Medicine Animal Studies Committee (IACUC #A-3381-01, Approval #20160092) and followed guidelines from the National Institutes of Health Guide for the Care and Use of Laboratory Animals. All efforts were made to minimize animal discomfort and reduce the number of animals used.

### Animals and surgery

**Fig 1:**
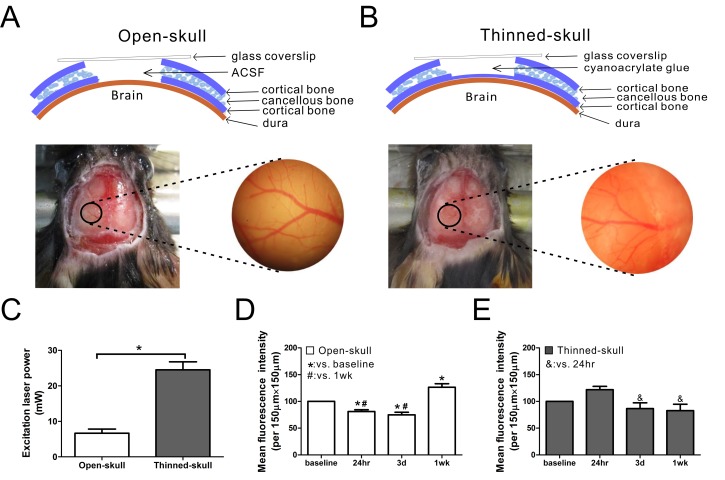
Schematic diagram and image analysis of open-skull technique and thinned-skull techniques for in vivo imaging of astrocytes in GFAP-GFP mice. (A,B) The mouse skull consists of two thin layers of cortical bone and a thick layer of cancellous bone. In the open-skull technique, all three layers of skull are removed and the exposed dura is coated with ACSF and covered with a glass coverslip over the skull window (A). In the thinned-skull technique, the skull is carefully thinned to the inner cortical bone to about 20 μm in thickness. The thinned skull is coated with a layer of cyanoacrylate glue and covered with a glass coverslip over the skull window (B). In both techniques, a round area of skull (~2 mm in diameter, marked with black circle) over the left somatosensory neocortex is removed (A) or thinned (B). Vasculature images captured after surgery help to ensure repeated observation from the same areas. (C) About 7 mW excitation laser power was needed for good image quality in the open-skull technique but much higher laser power (~25 mW) was required in thinned-skull technique to provide a similar image intensity (*p<0.05 by t-test, n = 6 per group). (D) In the open-skull technique, mean fluorescence intensity decreased 24 hr and 3 d after surgery but increased at 1wk (*,# p<0.05 by one-way ANOVA with Tukey post test, n = 6 per group). (E) In contrast, no significant change of mean fluorescence intensity was observed in the thinned-skull technique over time compared with baseline (p>0.05), although there was a slight decrease in intensity at 3 d and 1 wk compared to 24 hr (& p<0.05 by one-way ANOVA with Tukey post-test, n = 6 per group).

Two-to-three month old (~20 g) male GFAP-GFP transgenic mice with a mixed background, expressing enhanced green fluorescent protein (GFP) under a GFAP promoter were bred, originally obtained from Jackson Laboratory. Animal surgeries were performed using aseptic procedures as previously reported with minor modification [13,14]. Two surgery techniques, open-skull and thinned-skull technique, were performed (See S1 Methods for details). Briefly, mice were anesthetized with isoflurane and held in a custom-made stereotaxic device, which could be mounted to the microscope stage. A heating pad was used to maintain body temperature while under anesthesia. In open-skull surgery, a round cranial window (~2 mm in diameter) was first drilled in the skull with the center of the window approximately 3 mm posterior to bregma and 2 mm lateral to midline. All three layers of the skull were removed and the exposed dura was coated with ACSF (Artificial cerebrospinal fluid: 125 mM NaCl, 5 mM KCl, 2 mM CaCl_2_, 2 mM MgSO_4_, 10 mM Glucose, 10 mM HEPES, pH = 7.4) and then covered with a glass coverslip (#1 in thickness, 5 mm in diameter) over the window (Fig 1A). In thinned-skull surgery, the skull (a round area of ~2 mm in diameter) was carefully thinned to the inner cortical bone to about 20 μm in thickness (as measured optically using a dye on the skull surface in some experiments). The thinned skull was coated with a layer of cyanoacrylate glue (Krazy Glue, Elmer’s Products) and then covered with a glass coverslip (#1 in thickness, 5 mm in diameter) over the thinned-skull (Fig 1B). Cyanoacrylate glue and dental cement (SNAP, Parkwell inc) were applied around the edges of the coverslip to stabilize the coverslip to the skull. In some experiments, repeated thinning was performed 2 weeks after the first thinning using similar methods as above. Anti-inflammatory drugs were not used, given concerns about their effects on astrocytes, which may confound or mask the purpose of the imaging studies.

### Two-photon imaging

Images of astrocytes in neocortex were obtained through the cranial window using a two-photon microscope (LSM 510; Zeiss, Thornwood, NY) with a water immersion objective (Zeiss, 40×, 0.8 numerical aperture (NA), IR-adjusted, Zeiss). A Titanium-Sapphire pulsed infrared laser (Coherent, Santa Clara, CA) was used to stimulate GFP at 900 nm. The excitation laser power was manually calibrated to obtain an optimized image determined by saturation of the range indicator in LSM M510 software and was measured below the objective lens using a Coherent Field Master power/energy meter (Coherent, Santa Clara, CA). Low-magnification images approximate 50 to 100 μm below the neocortical surface were first obtained to identify regions with GFP positive astrocytes. At higher magnification (3× digital zoom), z-stacks of 6 to 10 images with 1 μm steps were taken. Individual images were acquired at 12 bits with frame averaging (2–4 times). Surface vasculature images captured by digital camera were used to identify the same astrocyte for time-lapse imaging at various times (baseline, 24 hr, 3 d, and 1 wk after surgery) (Fig 1A and 1B). Baseline images were obtained within 30 minutes of completion of surgery. The same excitation laser power and acquisition settings (e.g., detection gain, amplifier offset, amplifier gain) were maintained in individual animals at different time points for direct comparison. All mice included for analysis were successfully followed for one week observation. Mice that developed obvious complications (e.g. damaged dura, bleeding or severe clouding over the cranial window) during the observation period were excluded from analysis. In addition, on sequential imaging, mice that had a greater than 50% reduction in mean fluorescence intensity compared to baseline images were excluded. About 50% of mice met these exclusion criteria for both the thinned skull and open skull procedures.

### Post hoc image analysis

Post hoc image analysis was performed using LSM 5 Image Examiner software (Zeiss) and ImageJ software (NIH) in a blinded fashion to evaluate the changes in image intensity, astrocyte number, and morphological features of astrocytes over time. A standard area of 150 μm×150 μm was chosen as the region of interested (ROI) for each mouse, and the same ROI was analyzed at different time points. Image intensity was measured as mean GFP fluorescence intensity under the same excitation laser power and acquisition settings, with follow-up images over time normalized to the baseline image. Astrocyte number was counted in the same ROI at different time points. Morphological features of astrocytes were assessed with respect to total astrocyte size (including processes) and soma size, based on area calculations from the projected Z-stacks (S1 Fig).

### Immunohistochemistry

GFAP-GFP transgenic mice were anesthetized with isoflurane during cardiac perfusion of PBS followed by 4% paraformaldehyde (PFA, Electron Microscopy Science). Brains were then dissected out and post-fixed in 4% PFA overnight at 4°C followed by 30% sucrose dehydration. Coronal brain sections (45 μm in thickness) were prepared by frozen sectioning, and were blocked in PBS solution with 10% goat serum/1% BSA/0.3% Triton X-100 for 1 hour at room temperature. Glial fibrillary acid protein (GFAP) is a specific marker for astrocytes. The brains sections were incubated in the primary antibody (anti-GFAP, mouse, 1:2000, Cell Signaling) overnight at 4°C, and secondary antibody (CY3 goat-anti-mouse, 1:1000) at room temperature for 4 hr. For GFAP positive astrocyte counting, confocal images were taken with Zeiss LSM 5 PASCAL system coupled to Zeiss Axiovert 200 microscope. Comparable sections from different mice were chosen for comparison. A 0.3 mm^2^ area of each side of cerebral cortex was analyzed for GFAP positive astrocyte counting in a blinded fashion.

### Statistics

All data were presented as mean ± SEM. Statistical analysis was performed using GraphPad Prism 5 software. Student’s t test was used for comparison of laser power between groups. One-way analysis of variance (ANOVA) with the Tukey’s multiple comparison post-tests was used for comparison of mean fluorescence intensity, astrocytes number, the size of astrocytes and its soma, and soma-to-astrocyte ratio among different groups. P < 0.05 was set as statistical significance.

## Results

### Comparison of imaging properties between open-skull technique and thinned-skull techniques

We utilized and compared the open-skull and thinned-skull techniques with repeated 2PLSM of live astrocytes in neocortex of GFAP-GFP transgenic mice. In both methods, about 50% of mice were excluded from analysis due to dura damage, excessive bleeding, opacity of the initial baseline images, or a greater than 50% reduction in mean fluorescent intensity in follow-up images compared with baseline. The open-skull technique, in which all three layers of bone are removed, provides unimpeded optical access to the brain (Fig 1A). By comparison, in the thinned-skull technique, the remaining portion of inner cortical bone represents a partially opaque barrier to imaging (Fig 1B). As a result, a significantly higher excitation laser power was required to obtain comparable image intensity with the thinned-skull technique compared with the open-skull technique (Fig 1C). To monitor for changes in image intensity over time, the same laser power and acquisition settings were maintained in individual animals at different time points after surgery for direct comparison. In the open-skull technique, the image intensity decreased at 24 hr and 3 d, but increased at 1 wk after surgery, compared with baseline (Fig 1D). In contrast, with the thinned-skull technique, the image intensity did not change significantly at 24 hr, 3d, or 1 wk after surgery compared with baseline, although it decreased slightly at 3 d and 1 wk compared with 24 hr (Fig 1E). After 1 week, there was a significant decrease in image intensity with the thinned skull method (S2 Fig). However, repeated thinning was able to restore the previous image intensity (S2A–S2J Fig).

### Comparison of astrocyte number and morphology between open-skull and thinned-skull techniques

**Fig 2:**
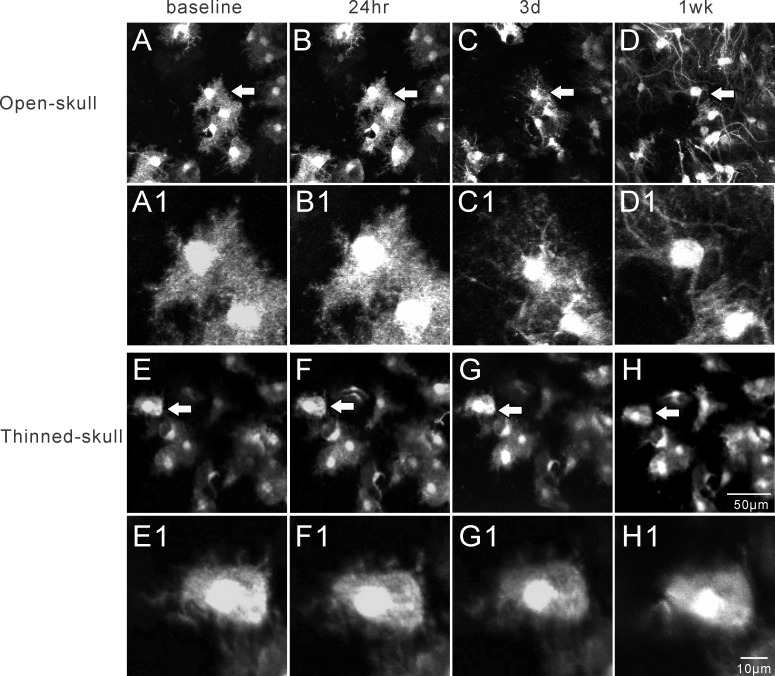
Representative in vivo images of astrocytes after open-skull technique (A-D) and thinned-skull technique (E-H) over a 1wk period. The arrows in figures A-H denote the astrocytes that are enlarged as in figure A1-H1, respectively. Astrocytes typically have a characteristic bushy appearance consisting of thin processes as shown in A1 and E1. After open-skull surgery, the size of astrocytes and their somas decreased at 3 d and 1 wk, but the number of astrocytes increased at 1 wk. Three days after open-skull surgery, the astrocytes started to lose their classical bushy appearance with fine processes and develop extension and hypertrophy of individual processes (C1, D1). No significant changes were observed in astrocytes after thinned-skull surgery over 1wk period (E-H, E1-H1).

**Fig 3:**
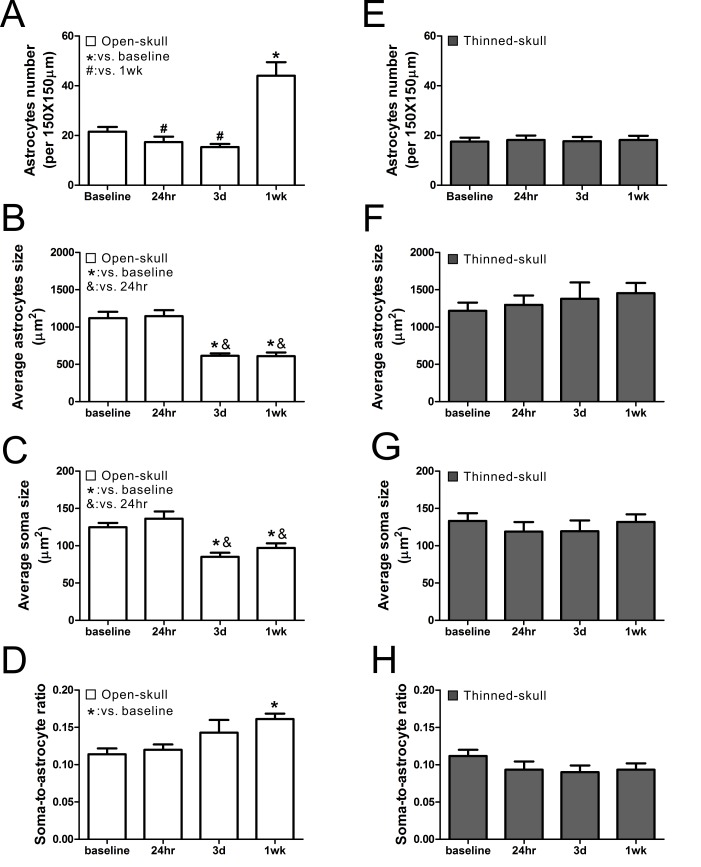
Comparison of astrocyte number and morphological features between open-skull technique (A-D) and thinned-skull technique (E-H). (A) In the open-skull technique, the number of astrocytes decreased at 24 hr and 3 d then increased at 1wk after surgery. (B-D) The size of astrocytes and their somas decreased at 3 d and 1 wk after surgery (B, C), while the ratio of the soma to astrocyte size increased at 1wk after surgery (D). (E-H) In the thinned-skull technique, no significant change was observed in astrocyte number, astrocyte or soma size, and the soma-to-astrocyte ratio at all observed time points after surgery. *,#,& p<0.05 by one way ANOVA with Tukey's post-test (n = 6 per group).

The open-skull technique, but not the thinned-skull technique, has been reported to induce astrogliosis as assayed by conventional histological methods [10,11], but this has not been studied directly by live imaging of astrocytes in vivo. We collected images and measured changes in astrocyte number, total astrocyte size, and astrocyte soma size, over time with both methods (See Fig 2 for representative images). Astrocyte number did not change at 24 hr and 3 d, but significantly increased at 1wk after open-skull surgery (Fig 3A) and persisted for at least 4–8 weeks (S3A Fig). By comparison, no significant changes in astrocyte number were observed at 24 hr, 3 d, or 1 wk after thinned-skull surgery (Fig 3E). Both total astrocyte size (Fig 3B) and soma size (Fig 3C) decreased significantly at 3 d and 1wk after open-skull surgery, while the soma-to-astrocyte ratio increased significantly at 1 wk after open-skull surgery (Fig 3D). In addition, protoplasmic astrocytes lost their classic bushy appearance, with individual processes becoming more prominent and extensive (see Fig 2D1). These morphological and proliferative changes consistent with astrogliosis were also present when a recovery period of 2–4 weeks was allowed after open-skull surgery prior to initial imaging (S3B and S3C Fig). In contrast, none of these morphological properties changed significantly over one week after thinned-skull surgery (Fig 3F–3H). Furthermore, beyond one week, repeated thinning did not show any evidence of astrogliosis based on astrocyte size and number (S2K–S2N Fig). However, at baseline, the open-skull method did appear to have a higher resolution than the thinned-skull method for imaging individual, fine astrocytic processes (Fig 2A1 and 2E1).

### Confirmation of astrogliosis by immunohistochemistry

**Fig 4:**
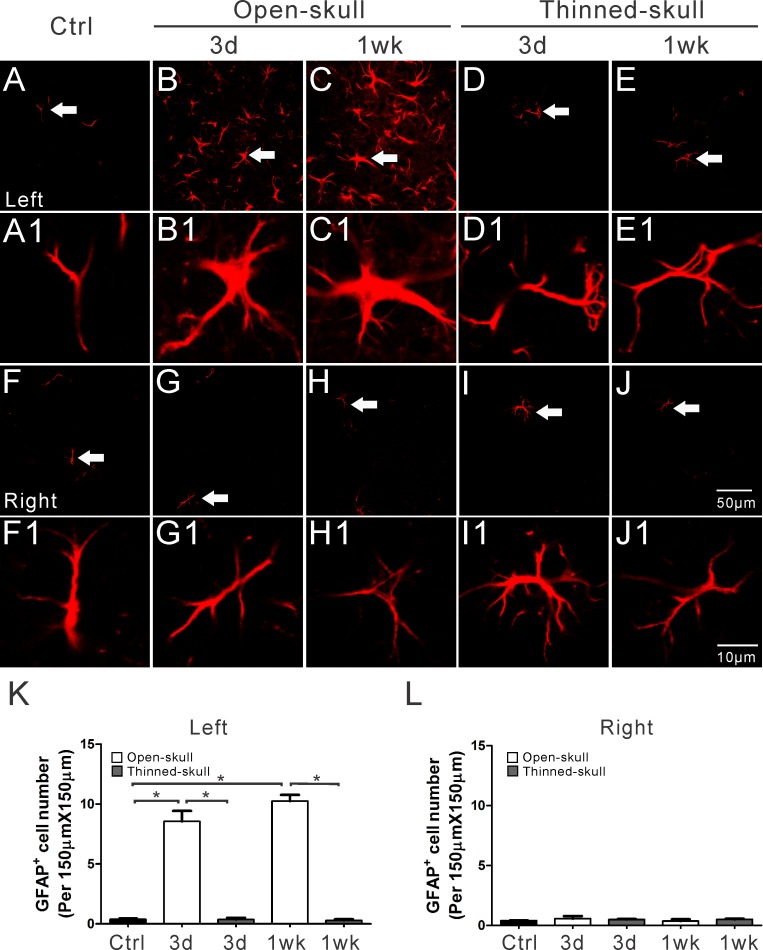
Immunohistochemical staining of GFAP in fixed tissue sections following open-skull and thinned-skull surgery in GFAP-GFP transgenic mice. Expression of GFAP (red) is a prototypical immunohistochemical marker of reactive astrocytes. GFAP positive astrocytes increased massively on the surgery side (left side) of the neocortex at 3 d and 1 wk after open-skull surgery (B, C) but not after thinned-skull surgery (D, E). Minimal GFAP positive astrocytes occurred on the contralateral side (right side) (G-J) and in control mice (A, F). The arrows in figures A-J denote the astrocytes that are enlarged as in figure A1-J1, respectively. Summarized data (K, L) indicate significantly more GFAP positive astrocytes in the left neocortex at 3 d and 1 wk in open-skull mice than in thinned-skull mice or in control mice (K), with no significant difference in the right neocortex (L). *p<0.05, by one way ANOVA with Tukey's post-test (n = 6 per group).

To control for the possibility that the changes in astrocyte number and size after open-skull surgery was an artifact of the changes in image intensity, in separate experiments we performed conventional immunohistochemical staining of GFAP for astrocytes on fixed brain sections at different time points after surgery. Consistent with the in vivo imaging, GFAP-positive cell number did not change over time after thinned-skull surgery, but increased significantly at 1wk after open-skull surgery (Fig 4). However, in contrast to the in vivo imaging, GFAP-positive cell number also increased significantly at 3d after open-skull surgery in the fixed sections. The increase in astrocyte number with open-skull, but not thinned-skull, methods was confirmed by two different astrocyte labeling methods: GFP-transgene expression and GFAP immunolabeling (S4 Fig), although these two methods labeled different subpopulations of astrocytes.

## Discussion

In vivo imaging has become an important method for investigating normal brain function and neurological disease. The use of 2PLSM in mice with genetically-encoded fluorescent proteins has revolutionized the study of dynamic cellular and subcellular structures in the living brain. For example, in vivo imaging of neurons has revealed a degree of plasticity to dendrites and dendritic spines that was unappreciated with conventional pathological methods [15,16]. Dendritic spine turnover decreases over the course of development and may also correlate with learning [16,17,18]. Furthermore, pathological insults, such as stroke or seizures, causes acute changes in neuronal structure that can be detected on a very rapid time scale with time-lapse in vivo imaging [14,19,20]. However, differences in imaging methods, particularly the use of open-skull versus thinned-skull techniques, have led to significantly different results in the degree of dendritic plasticity, with the open-skull method having a much higher degree of dendritic spine turnover compared with the thinned-skull method [10]. This difference has been attributed to increased perturbation and inflammation that may occur, as partially reflected by astrogliosis detected on pathological analysis, with the open-skull method. However, a direct analysis of the evolution of in vivo imaging of astrocytes over time and a comparison of in vivo astrocyte imaging between open-skull and thinned-skull methods has not been previously reported. In the present study, we demonstrate that the thinned-skull method appears to be preferable for imaging astrocytes for most situations, as the open-skull method induces astrogliosis, which is not observed with the thinned-skull method.

In the open-skull technique, given the unimpeded optical access to the brain, a relatively low excitation laser power (~7 mW) was required to obtain adequate fluorescence intensities compared with the thinned-skull method. In addition, the spatial resolution of the fine astrocytic processes was slightly better with the open-skull method. However, the open-skull method may cause more mechanical disruption of brain parenchymal and meningeal structures and introduce foreign particles to the brain, which may in turn produce inflammatory changes. This may increase opacity of the window, which is consistent with our finding that the image intensity decreased at 24 hr and 3d after open-skull surgery. Thus, it is often recommended that imaging studies start at least 1–2 weeks after open-skull surgery. However, we actually observed an increase in image intensity at 1 week after the surgery, which is likely due to reactive astrogliosis and upregulation of GFAP expression, leading to a direct increase in fluorescence intensity in the GFAP-GFP mice. In contrast, the image intensity stayed constant over 1 week observation after thinned-skull surgery, although a higher absolute laser power (~25 mW) was required due to the residual inner cortical bone barrier. On the other hand, the remaining inner cortical bone may also provide protection against inflammation which may improve image quality. We utilized the newer reinforced thinned skull method with a fused transparent cement and glass window [13], and eventually did see decreases in image intensity beyond one week, possibly reflecting partial bone regrowth. In contrast, repeated thinning [12] was able to restore the previous image intensity and may represent the better method for longer-term astrocyte imaging.

Under normal physiological conditions, astrocytes maintain a relatively stable number and morphology, with a bushy appearance and thin processes. No significant changes were detected in astrocytes morphological features after thinned-skull surgery at all observed time points, including after repeated thinning. In contrast, the open-skull surgery led to significant astrogliosis, as evident by both an increase in astrocyte number and changes in size and morphology with time-lapse in vivo imaging. As reported by others [10,11], we confirmed by conventional immunohistochemical staining of GFAP on fixed brain sections that reactive astrogliosis occurs after open-skull, but not with thinned-skull, surgery. Compared with the in vivo imaging, fixed tissue studies showed an overall lower density of astrocytes, likely due to differences in sampling (superficial vs. deeper layers) and labeling method (transgenic expression vs. immunohistochemical labeling), but did still show a significant increase in astrocyte number in the open-skull method. Astrogliosis following open-skull surgery may persist for at least 4 weeks as monitored by in vivo imaging, even after allowing a two-four week recovery period from surgery (S3 Fig), and with conventional histology [10]. Thus, the standard approach of waiting to image after open-skull surgery may not be adequate to eliminate astrogliosis as a confounding factor in many in vivo imaging studies. Anti-inflammatory drugs are often used with the open-skull method, which could potentially reduce this astrogliosis. However, the use of anti-inflammatory drugs could confound or mask potential effects or changes in astrocytes that are the subject of the imaging study, so we did not use them in this study and suggest that they not be used for other imaging studies focused on astrocyte modulation. Given the importance of astrocytes in both physiological and pathological processes, this study suggests that the thinned-skull method is preferred for in vivo imaging of astrocytes, especially when monitoring for astrogliosis or following changes in astrocyte number or shape over time. However, the open skull method may be advantageous for assessing acute changes in fine astrocytic processes, such as dynamics of endfeet with neurovascular coupling.

## Conclusions

The thinned-skull method provided stable images of astrocytes in vivo over at least one week, and longer when using repeated thinning, with no evidence of astrogliosis. In contrast, the open-skull method resulted in significant changes in image intensity and induced astrogliosis. The thinned-skull method is the preferred approach for in vivo imaging of astrocytes.

## Supporting Information

S1 Fig Measurement of astrocyte and soma size. Morphological features of astrocytes were assessed with respect to total astrocyte size (including processes) and soma size, based on area calculations from projected Z-stacks. Areas were measured using ImageJ software. The brightness of the in vivo image was manually adjusted for optimal contrast. To measure the area of astrocyte soma (excluding branches/fine processes) and total area, lines were drawn as shown.
(TIF)

S2 Fig Decreased image intensity 2 weeks after thinned-skull method, but restoration with repeated thinning. (A) Schedule for imaging and repetitive thinning for assessment of the thinned-skull method beyond week. Images were obtained for two weeks after using the initial thinned skull surgery. As image intensity significantly decreased at 2 weeks, a second thinning was then performed. (B-I). Representative in vivo images of astrocytes with the repeated thinned skull-method, involving a second thinning at 2 weeks after the initial thinning. The arrows in figures B-I denote the astrocytes that are enlarged as in figure B1-I1, respectively. The effect of repeated thinning of the skull was assessed on image intensity (J) and astrocyte number and size (K-N). (J) Image intensity decreased at 2 weeks after the initial thinning, but repeated thinning restored image intensity at least for another week. (K-N). With repeated thinning, no significant change was observed in astrocyte number, astrocyte or soma size, and the soma-to-astrocyte ratio at all observed time points after surgery. * p<0.05 by one way ANOVA with Tukey's post-test (n = 6 per group).
(TIF)

S3 Fig Astrogliosis with the open skull method persists for greater than two weeks. A) Extension of time-lapse in vivo imaging beyond the initial one week period demonstrates that the astrogliosis that occurred at 1 week after open-skull surgery persisted for at least 7 weeks. B,C) In other animals, surgery was performed, but the first images were not obtained until 2 (B) or 4 (C) weeks after surgery (no baseline or follow-up images prior to 2 weeks). Despite this 2–4 week waiting period after surgery, astrogliosis appeared present with the first imaging session and persisted.
(TIF)

S4 Fig Comparison of astrocyte expression in fixed sections following open-skull and thinned-skull surgery by two different astrocyte labeling methods. Astrocytes were labeled by GFAP-GFP transgene expression (green) and GFAP immunohistochemical staining (red) in the same sections following open-skull and thinned skull surgery on the left side (no surgery on contralateral right side). Double labeled cells are shown in yellow, indicating astrocytes labeled by both methods. Minimal labeling occurs by either labeling method on the contralateral right side for both open-skull and thinned-skull surgery (F-J, I). On the surgery side (left), astrocytes were increased at 3 days and 1 week after open-skull, but not thinned-skull, surgery with both labeling methods (A-E, K), but interestingly the two labeling methods primarily labeled two different subsets of astrocytes with only modest overlap/double labeling.
(TIF)

S1 Methods Supplementary methods for open-skull and thinned-skull surgery. (DOCX)
