# Supplementary figures and images for: In Vivo Two-Photon Imaging of Astrocytes in GFAP-GFP Transgenic Mice
Source: PLoS One. 2017 Jan 20;12(1):e0170005. doi: 10.1371/journal.pone.0170005 (PMC5249218; doi:10.1371/journal.pone.0170005)

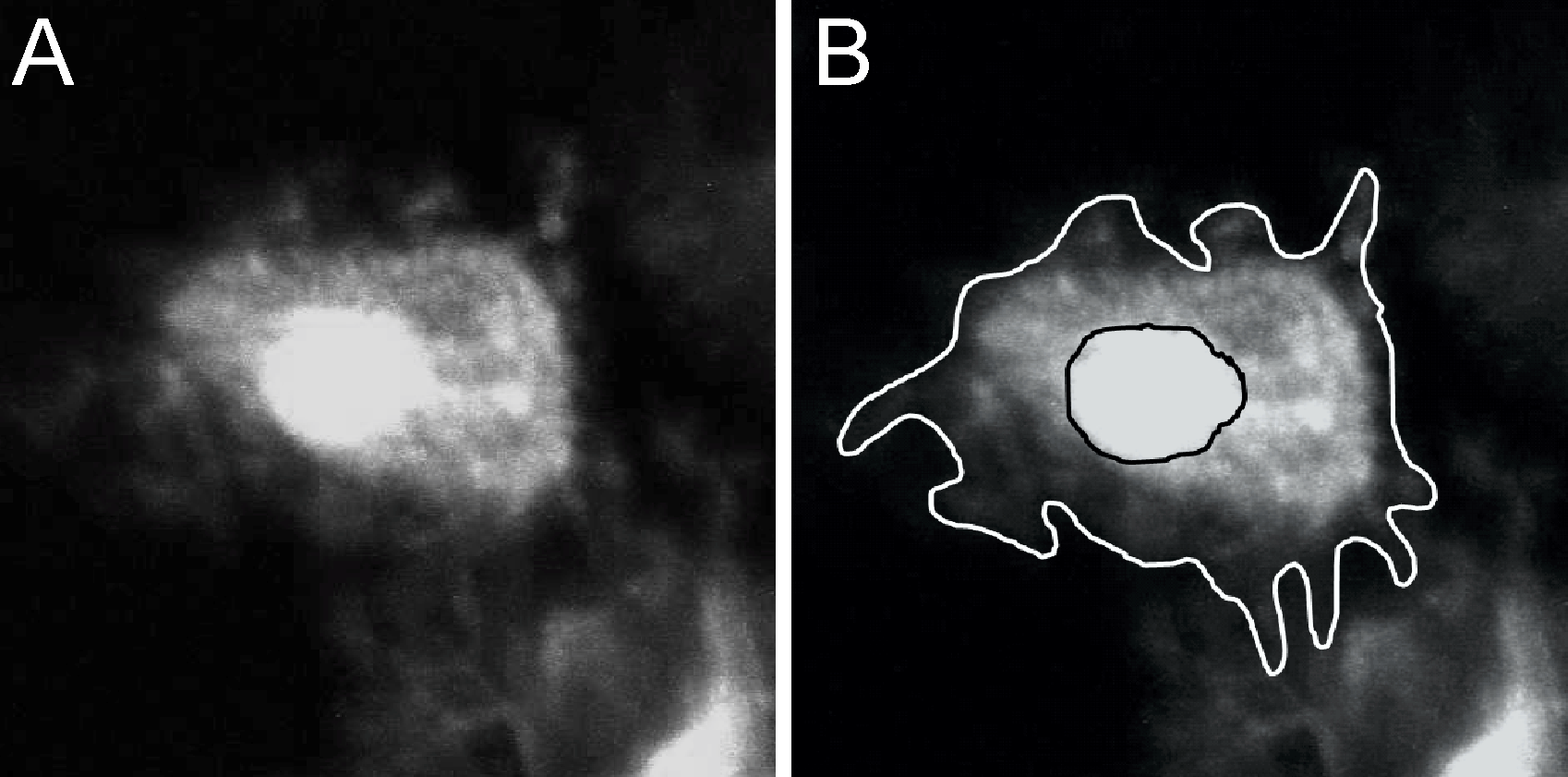

Supplement: S1 Fig — Morphological features of astrocytes were assessed with respect to total astrocyte size (including processes) and soma size, based on area calculations from projected Z-stacks. Areas were measured using ImageJ software. The brightness of the in vivo image was manually adjusted for optimal contrast. To measure the area of astrocyte soma (excluding branches/fine processes) and total area, lines were drawn as shown. (TIF) [file pone.0170005.s001.tif]

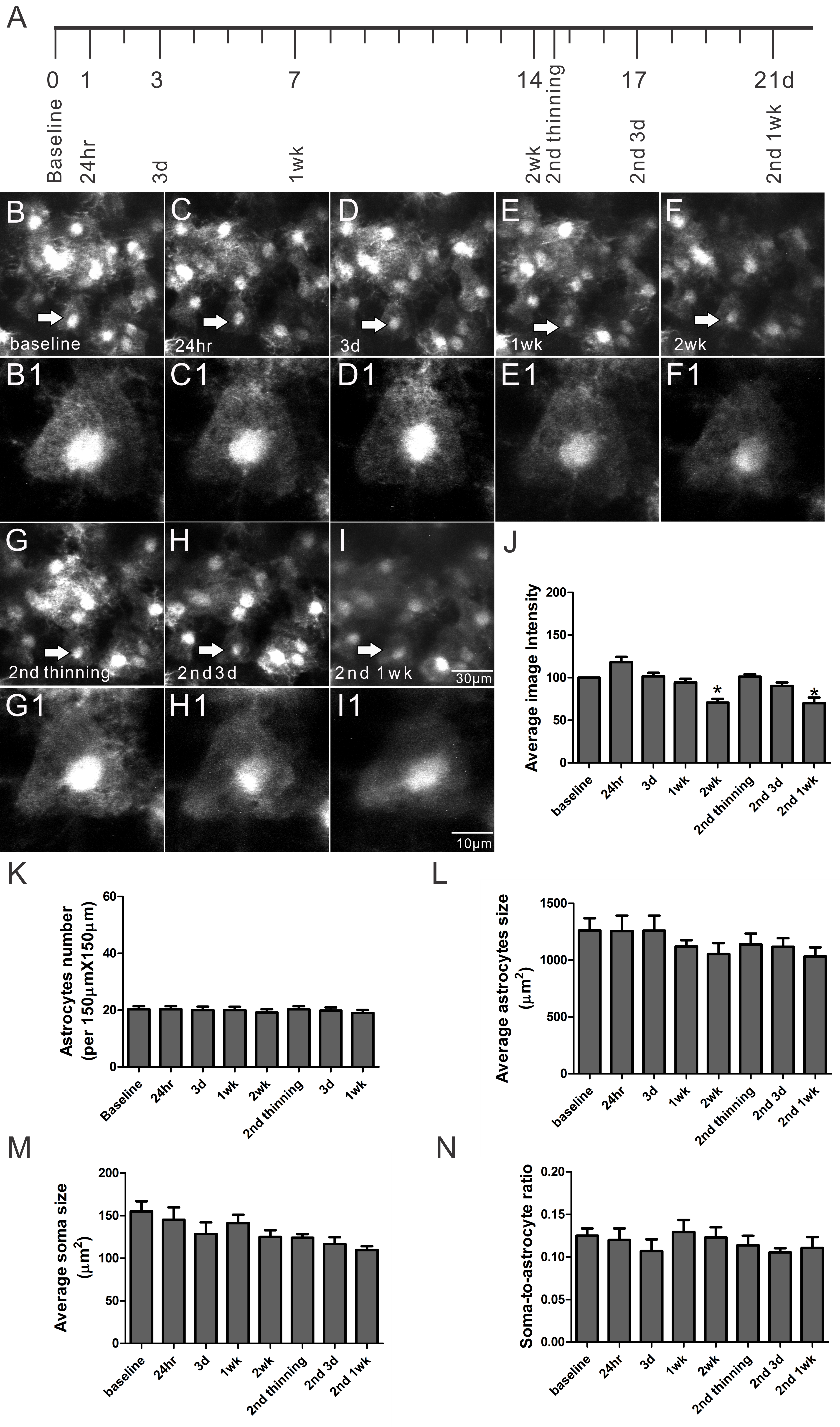

Supplement: S2 Fig — (A) Schedule for imaging and repetitive thinning for assessment of the thinned-skull method beyond week. Images were obtained for two weeks after using the initial thinned skull surgery. As image intensity significantly decreased at 2 weeks, a second thinning was then performed. (B-I). Representative in vivo images of astrocytes with the repeated thinned skull-method, involving a second thinning at 2 weeks after the initial thinning. The arrows in figures B-I denote the astrocytes that are enlarged as in figure B1-I1, respectively. The effect of repeated thinning of the skull was assessed on image intensity (J) and astrocyte number and size (K-N). (J) Image intensity decreased at 2 weeks after the initial thinning, but repeated thinning restored image intensity at least for another week. (K-N). With repeated thinning, no significant change was observed in astrocyte number, astrocyte or soma size, and the soma-to-astrocyte ratio at all observed time points after surgery. * p<0.05 by one way ANOVA with Tukey's post-test (n = 6 per group). (TIF) [file pone.0170005.s002.tif]

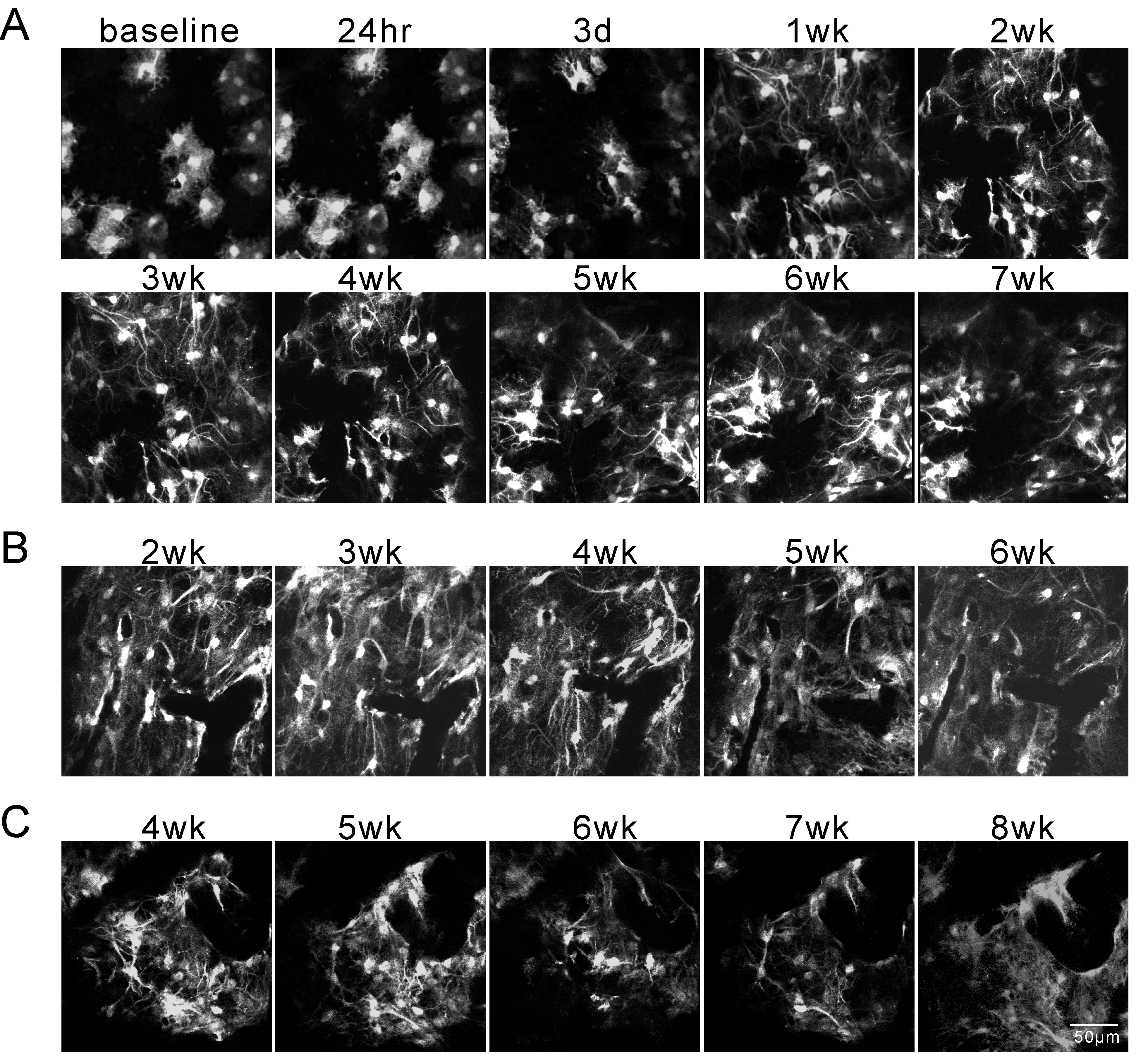

Supplement: S3 Fig — A) Extension of time-lapse in vivo imaging beyond the initial one week period demonstrates that the astrogliosis that occurred at 1 week after open-skull surgery persisted for at least 7 weeks. B,C) In other animals, surgery was performed, but the first images were not obtained until 2 (B) or 4 (C) weeks after surgery (no baseline or follow-up images prior to 2 weeks). Despite this 2–4 week waiting period after surgery, astrogliosis appeared present with the first imaging session and persisted. (TIF) [file pone.0170005.s003.tif]

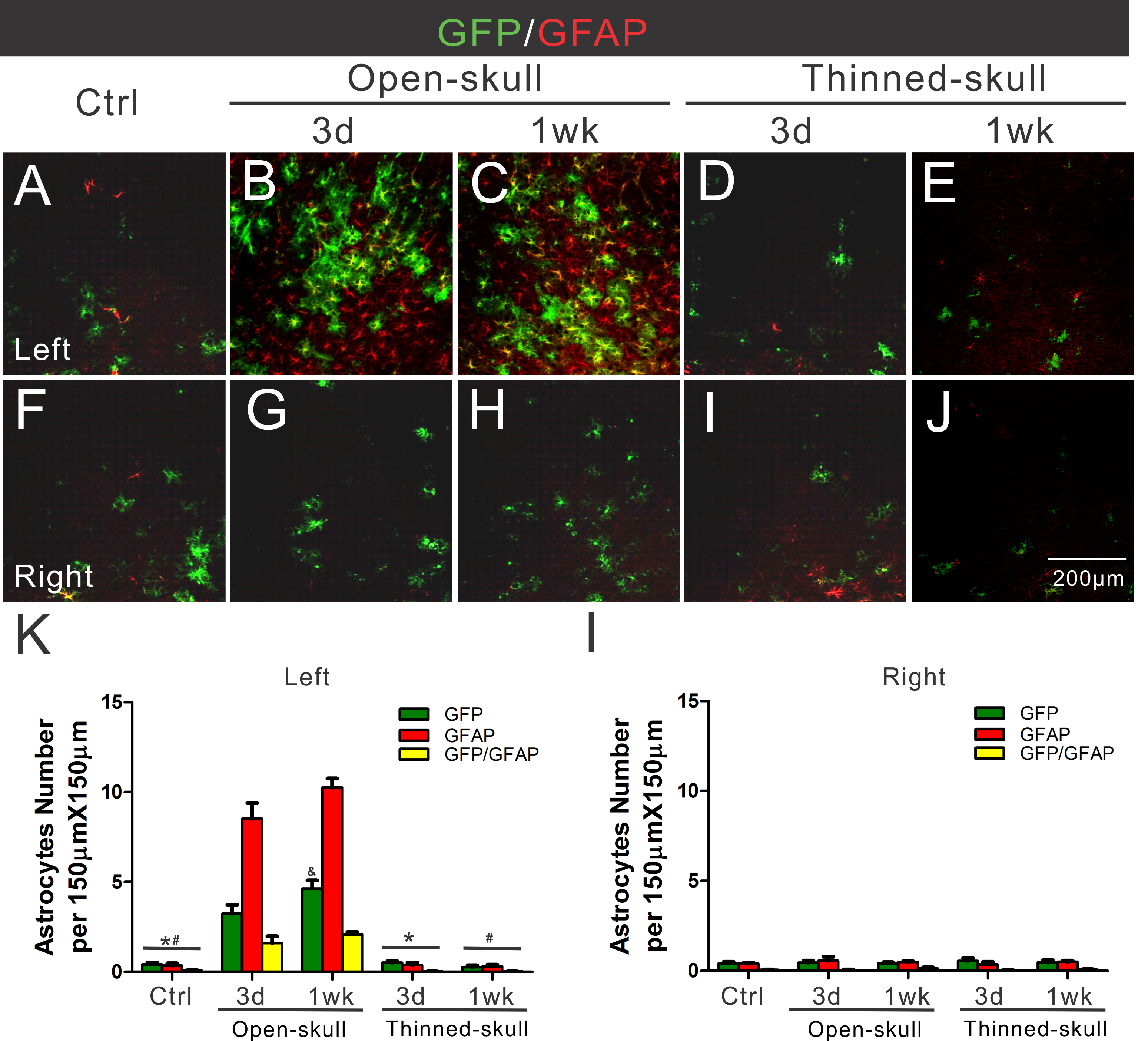

Supplement: S4 Fig — Astrocytes were labeled by GFAP-GFP transgene expression (green) and GFAP immunohistochemical staining (red) in the same sections following open-skull and thinned skull surgery on the left side (no surgery on contralateral right side). Double labeled cells are shown in yellow, indicating astrocytes labeled by both methods. Minimal labeling occurs by either labeling method on the contralateral right side for both open-skull and thinned-skull surgery (F-J, I). On the surgery side (left), astrocytes were increased at 3 days and 1 week after open-skull, but not thinned-skull, surgery with both labeling methods (A-E, K), but interestingly the two labeling methods primarily labeled two different subsets of astrocytes with only modest overlap/double labeling. (TIF) [file pone.0170005.s004.tif]
