# Supplementary material for: In Vivo Two-Photon Imaging of Astrocytes in GFAP-GFP Transgenic Mice
Source: PLoS One. 2017 Jan 20;12(1):e0170005. doi: 10.1371/journal.pone.0170005 (PMC5249218; doi:10.1371/journal.pone.0170005)
Supplement: S1 Methods — (DOCX) [file pone.0170005.s005.docx]

**S1 Methods. Supplementary methods for open-skull and thinned-skull surgery.
Open-skull surgery:**

1. Anesthetize the mouse with isoflurane (4% for induction, 2% for surgery).
2. Hold the mouse in a custom-made stereotaxic device.
3. Use a heating pad to maintain body temperature at 37C° under anesthesia.
4. Cover the eyes with eye ointment to retain moisture.
5. Shave the head with a small electrical razor and disinfect the shaved area with Betadine.
6. Remove the skin between the eyes to the back of the head and scrape out the periosteum and adjacent connective tissue.
7. Use a high-speed micro-drill with a new drill bit (0.7 mm in diameter) to drill a round cranial window (~2 mm in diameter) in the skull with the center of the window approximately 3 mm posterior to bregma and 2mm lateral to midline.
8. Apply cold saline regularly to prevent the frictional heating.
9. Carefully lift the flap of bone using a fine forceps
10. Gently rinse the exposed dura with ACSF to remove any bone debris.
11. Use a small piece of absorbable gelatin sponge (Gelfoam, Pharmacia & Upjohn) to stop minor bleeding.
12. Wet the exposed dura with a small drop of ACSF and dry the skull surface around the window thoroughly.
13. Place a circular glass coverslip (#1 thickness, 5 mm in diameter) on the top of the exposed dura, flush with the skull.
14. Seal the coverslip to skull with a thin layer of cyanoacrylate glue to stabilize the coverslip.
15. Place dental cement (SNAP, Parkwell inc) around the coverslip and all the exposed skull to further stabilize the coverslip.
16. Finally apply a thin layer of dental cement around the edge of the cover slip to provide a reservoir for holding water for immersion objective.
17. Start baseline imaging immediately after the surgery.

**Thinned-skull surgery**

- 1. Same as open-skull surgery

1. Use a high-speed micro-drill with a drill bit (0.7 mm diameter) to thin a round area (~2 mm in diameter) in the external cortical bone and cancellous bone with the center of the area approximately 3 mm posterior to bregma and 2 mm lateral to midline.
2. Apply cold saline regularly to prevent the frictional heating during thinning.
3. Continue to thin the inner cortical bone with a finer drill bit (0.5 mm diameter) until about 20 um bone is left.
4. Gently rinse the thinned area with ACSF to remove any bone debris.
5. Use a small piece of absorbable gelatin sponge (Gelfoam, Pharmacia & Upjohn) to stop minor bleeding if necessary.
6. Dry the thinned area and surrounding skull thoroughly.
7. Apply a thin layer of cyanoacrylate glue over the thinned area
8. Place a circular glass coverslip (#1 thickness, 5 mm diameter) over the thinned area, flushing with the skull.
9. Place dental cement (SNAP, Parkwell inc) around the coverslip and all the exposed skull to further stabilize the coverslip.
10. Finally apply a thin layer of dental cement around the edge of the coverslip to provide a reservoir for holding water for immersion objective.
11. Start baseline imaging immediately after the surgery.
